# A Systematic Review of Nirmatrelvir/Ritonavir and Molnupiravir for the Treatment of Coronavirus Disease 2019

**DOI:** 10.1093/ofid/ofae497

**Published:** 2024-09-07

**Authors:** Alyson Haslam, Vinay Prasad

**Affiliations:** Department of Epidemiology and Biostatistics, University of California, San Francisco, San Francisco, California, USA; Department of Epidemiology and Biostatistics, University of California, San Francisco, San Francisco, California, USA

**Keywords:** clinical trial, COVID-19, molnupiravir, nirmatrelvir/ritonavir, randomized

## Abstract

**Background:**

To address the need for treatments for patients with coronavirus disease 2019 (COVID-19), 3 therapies have been given either full approval or Emergency Use Authorization. These were based on randomized data showing a reduction in deaths/hospitalization, but since then, circulating viral strains and population immunity have changed.

**Methods:**

We searched PubMed, Web of Science, Embase, and ClinicalTrials.gov for clinical trials testing nirmatrelvir/ritonavir and molnupiravir for COVID-19. We identified all trials testing nirmatrelvir/ritonavir and molnupiravir in patients with COVID-19 and assessed the pooled efficacy in a meta-analysis. We calculated pooled estimates of hospitalization and death in patients with COVID-19 and the number of studies with published/reported data.

**Results:**

Of the 23 studies found, 11 tested nirmatrelvir/ritonavir, 10 tested molnupiravir, and 2 tested both agents. The pooled estimate in reducing deaths and hospitalization for molnupiravir was 0.62 (95% confidence interval [CI], 0.15–2.53), and the pooled estimate for nirmatrelvir/ritonavir was 0.33 (95% CI, 0.03–3.35). The 1 nirmatrelvir/ritonavir trial that reported significant improvements tested people who were predominantly infected with earlier COVID-19 variants, whereas the 2 null trials were tested in people infected with more recent variants. The 2 positive molnupiravir trials included participants primarily with the Delta variant, whereas the null trials were tested later, against more recent variants.

**Conclusions:**

While early trial data show effectiveness of these therapies, the overall pooled effects are nonsignificant, suggesting that recommendations and use of approved oral COVID-19 treatment therapies need to be reevaluated in the context of current viral strains and population immunity.

The emergence of the severe acute respiratory syndrome coronavirus 2 (SARS-CoV-2) created an urgent need for treatments for individuals who have a high risk of serious outcomes from coronavirus disease 2019 (COVID-19). Three drugs have been approved by the US Food and Drug Administration (FDA) to reduce the risk of hospitalization and/or death for patients with mild to moderate COVID-19—nirmatrelvir/ritonavir, molnupiravir, and remdesivir. Nirmatrelvir/ritonavir and molnupiravir are oral formulations and may be more likely to be used in the outpatient setting, compared to remdesivir, which is administered intravenously, due to their ease of administration.

Based on randomized data showing reduced hospitalization/death, nirmatrelvir/ritonavir was given Emergency Use Authorization (EUA) on 22 December 2021 (fully approved on 25 May 2023) [1, 2], and molnupiravir was given EUA on 23 December 2021 [3]. The early approvals were based on trials that excluded participants who were vaccinated (Evaluation of Protease Inhibition for Covid-19 in High-Risk Patients [EPIC-HR]), had not been previously infected with SARS-CoV-2 (EPIC-HR), and/or were tested during earlier strains of SARS-CoV-2 (eg, Delta) that are no longer circulating (EPIC-HR and Evaluation of Protease Inhibition for Covid-19 in Standard-Risk Patients [EPIC-SR]) [4, 5]. Since then, other trials have been planned and initiated (eg, Randomised Evaluation of COVID-19 Therapy [RECOVERY]) [6]. These trials have been tested against more contemporary strains of the virus and have included patients who have been vaccinated and/or acquired immunity through virus exposure.

Lingering symptoms from SARS-CoV-2 infection have been reported by some, which has prompted additional studies to examine these therapies in the context of long COVID.

The evolving strains of SARS-CoV-2 and increasing population immunity, either through vaccination or infection, results in changing effectiveness of the approved drugs. We have seen this in other COVID-19 therapies, such as bamlanivimab, which had its EUA revoked due to decreased effectiveness [7]. Yet, these oral drugs continue to be approved and recommended [8]. We therefore sought to systematically review all trials testing nirmatrelvir/ritonavir or molnupiravir in the treatment of COVID-19 and to characterize the available evidence for these therapies.

## METHODS

We sought to systematically and comprehensively search all randomized trial data on FDA-approved oral drug therapies in treating mild to moderate COVID-19, including nirmatrelvir/ritonavir and molnupiravir. We searched PubMed using the search terms (nirmatrelvir and ritonavir) OR paxlovid AND COVID. We searched Embase using the terms ((“nirmatrelvir”/exp OR nirmatrelvir) AND (“ritonavir”/exp OR ritonavir) OR “paxlovid”’/exp OR paxlovid) AND (“COVID”/exp OR COVID). We searched Web of Science using the search terms (NIRMATRELVIR AND RITONAVIR) OR paxlovid AND COVID AND randomized). For the molnupiravir search, we searched PubMed using molnupiravir AND COVID. We searched Embase using (“molnupiravir”/exp OR molnupiravir) AND (“COVID”/exp OR COVID). We searched Web of Science using molnupiravir AND COVID AND randomized. We included all interventional studies testing nirmatrelvir/ritonavir or molnupiravir for COVID-19, including long COVID, and the study population could include either hospitalized or nonhospitalized patients.

We further searched ClinicalTrials.gov to find additional studies with unpublished data on this topic. The search criteria include COVID-19 in the “condition/disease” search box and either Paxlovid, nirmatrelvir/ritonavir, or molnupiravir in the “other” search box, restricted to interventional studies. Once trials were identified, we searched PubMed, Google, and Google Scholar, using trial identification number and/or trial name to find published (peer-reviewed or not) result data.

All searches were made 12 December 2023. Embase and PubMed searches were restricted to randomized controlled trials. However, we later included a preprint publication on the RECOVERY trial, which was published after our initial search date [6]. We included studies reporting on the primary analysis of a randomized trial testing the efficacy of either the combination of nirmatrelvir and ritonavir or molnupiravir monotherapy. Studies needed to be in humans, and they needed to test 1 of the therapies against a standard of care at the time of the trial (ie, we excluded studies that were head-to-head, such as nirmatrelvir/ritonavir vs molnupiravir). Finally, studies needed to test the intervention in the general population (ie, we excluded studies in pregnant women or patients with kidney failure). We excluded nonrandomized studies (including reviews and commentaries), secondary analyses of randomized data, bioavailability studies, simulation studies, and cellular/in vitro studies. We also excluded studies that tested these therapies in preventing the incidence or transmission of COVID-19.

We abstracted trial data on dates of trial start and ending, phase, blinding status, number of patients (overall and in each treatment arm), primary outcome, study population, the number of people in each group with the outcome of hospitalization or death, predominant variant, vaccination status of participants, and trial registry number and name. From the trials on ClinicalTrials.gov, we noted whether the trials were terminated, the primary outcome, and the study start and estimated end date.

### Statistical Analysis

We calculated descriptive statistics for studies evaluating these therapies. All analyses were done using R statistical software, version 4.2.1. We used the “meta” package to calculate pooled relative risk of hospitalization and/or death for studies reporting the number of patients in each treatment arm with the outcome. We used a random-effects model to account for any between-study heterogeneity, and used the Mantel-Haenszel method to calculate the study weights. We used Knapp-Hartung adjustments in calculating confidence intervals (CIs) around the pooled effects. We used a restricted maximum likelihood estimator for τ^2^.

In accordance with 45 Code of Federal Regulations §46.102(f), this study was not submitted for University of California, San Francisco, institutional review board approval because it involved publicly available data and did not involve individual patient data. We adhered to Preferred Reporting Items for Systematic Reviews and Meta-Analyses (PRISMA) reporting guidelines.

## RESULTS

For nirmatrelvir/ritonavir, we found 517 results in Web of Science, 34 in Embase, and 9 in PubMed. For molnupiravir, we found 59 results in Web of Science, 34 in Embase, and 16 in PubMed. We found 93 relevant studies on ClinicalTrials.gov. After removing 85 duplicates from the search of publication databases and 31 duplicates from the ClincalTrials.gov search, we searched 584 records from the published literature and 62 records on ClinicalTrials.gov (Supplementary Figure).

We found 23 studies that tested these therapies in patients with COVID-19, which met our inclusion criteria. Eighteen tested COVID-19 treatments, and 5 tested long-COVID treatments. Characteristics of these trials are shown in Table 1, stratified by publication status, and in the Supplementary Table. Eleven tested nirmatrelvir/ritonavir, 10 tested molnupiravir, and 2 tested both agents. Most were phase 2 or 2/3 (n = 11 [47.8%]) or phase 3 or 4 studies (n = 9 [39.1%]), 1 (5.6%) was a phase 1 trial, and 2 did not indicate the phase. Ten (43.5%) were open label, 12 (52.2%) were blinded, and 1 did not indicate. The most common primary outcome was change in symptoms (n = 8 [34.8%]), followed by hospitalization/death (n = 6 [26.1%]), viral clearance (n = 5 [21.7%]), death (n = 2 [8.7%]), hospitalization (n = 1 [4.3%]), and quality of life (n = 1 [4.3%]). Omicron was the most common predominant variant (n = 8 [34.8%]), followed by Delta (n = 4 [17.4%]), Alpha (n = 2 [8.7%]), and Delta/Omicron (n = 1 [4.3%]), but the variant was not able to be determined in 8 studies (all without published trial data). Three trials were eventually terminated, and 6 trials were completed, according to ClinicalTrials.gov.

**Table 1.** Characteristics of Randomized Studies Evaluating the Efficacy of Nirmatrelvir/Ritonavir or Molnupiravir in Patients With Coronavirus Disease 2019 and Long COVID

Characteristic	No Published Data^a^	Published Data
No. of trials	9	14
Drug, No. (%)		
Molnupiravir	2 (22.2)	8 (57.1)
Nirmatrelvir/ritonavir	7 (77.8)	4 (28.6)
Both	0 (0.0)	2 (14.3)
Phase, No. (%)		
1	0 (0.0)	1 (7.1)
2	4 (44.4)	3 (21.4)
2/3	0 (0.0)	4 (28.6)
3	5 (55.6)	3 (21.4)
4	0 (0.0)	1 (7.1)
NI	0 (0.0)	2 (14.3)
Blind, No. (%)		
Blind	6 (66.7)	6 (42.9)
NI	0 (0.0)	1 (7.1)
Open	3 (33.3)	7 (50.0)
No. of participants, median (IQR)	…	278 (152–1130)
Predominant variant, No. (%)		
Alpha	0 (0.0)	2 (14.3)
Delta	0 (0.0)	4 (28.6)
Delta/Omicron	0 (0.0)	1 (7.1)
NI	8 (88.9)	0 (0.0)
Omicron	1 (11.1)	7 (50.0)
Country, No. (%)		
Belgium	0 (0.0)	1 (7.1)
Canada	1 (11.1)	0 (0.0)
China	0 (0.0)	3 (21.4)
India	0 (0.0)	1 (7.1)
Multiple	0 (0.0)	5 (35.7)
Norway	1 (11.1)	0 (0.0)
Russia	2 (22.2)	0 (0.0)
South Africa	1 (11.1)	0 (0.0)
Sweden	1 (11.1)	0 (0.0)
Thailand	0 (0.0)	1 (7.1)
United Kingdom	0 (0.0)	2 (14.3)
United States	3 (33.3)	1 (7.1)
Percentage vaccinated, median (IQR)	…	67.0 (0–92)
Primary outcome, No. (%)		
Death	0 (0.0)	2 (14.3)
Hospitalization	0 (0.0)	1 (7.1)
Hospitalization/death	2 (22.2)	4 (28.6)
Quality of life	1 (11.1)	0 (0.0)
Symptoms	6 (66.7)	2 (14.3)
Viral clearance	0 (0.0)	5 (35.7)
Status on ClincialTrials.gov, No. (%)		
Completed	3 (33.3)	3 (21.4)
Ongoing	6 (66.7)	8 (57.1)
Terminated	0 (0.0)	3 (21.4)

Abbreviations: IQR, interquartile range; NI, not indicated.

^a^Data were considered unpublished if we were unable to find any peer-reviewed publications, preprints, or conference abstracts reporting trial results, or if there were no results published on ClinicalTrials.gov.

**Figure 1.**
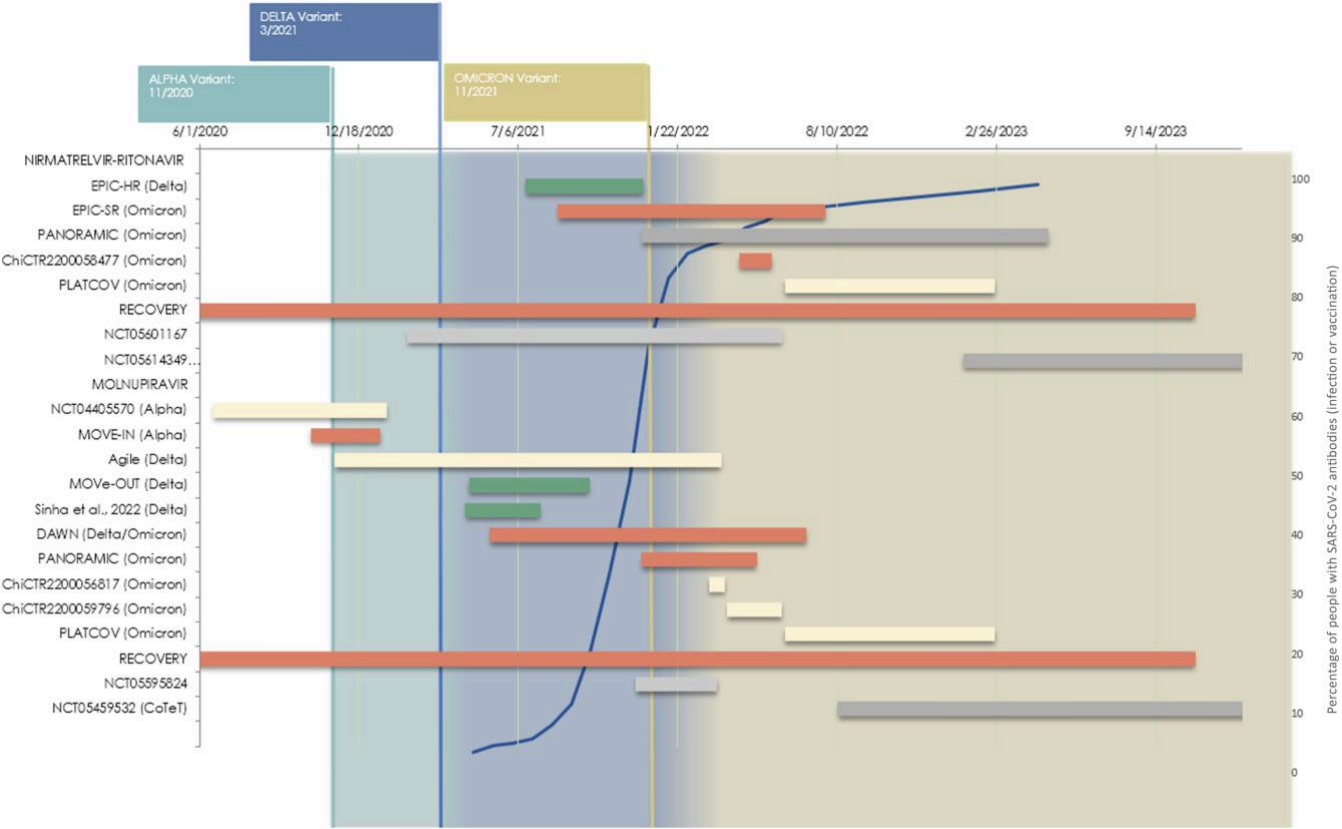
Timeline of trials testing nirmatrelvir/ritonavir and/or molnupiravir in patients with coronavirus disease 2019. Red indicates negative results for hospitalization and death, green indicates positive results for hospitalization and death, yellow indicates softer outcomes (eg, viral clearance), and gray indicates no published results. The width of the bar represents the time between the start and end of enrollment. The blue line in the background indicates the percentage of the population with severe acute respiratory syndrome coronavirus 2 (SARS-CoV-2) antibodies (https://COVID.cdc.gov/COVID-data-tracker/#nationwide-blood-donor-seroprevalence). Abbreviations: RECOVERY, randomised evaluation of covid-19 therapy; EPIC-SR, evaluation of protease inhibition for covid-19 in standard-risk patients; EPIC-HR, evaluation of protease inhibition for covid-19 in high-risk patients.

The studies examining these therapies, a timeline of each study's enrollment history, and their results are presented in Figure 1.

We were able to find reported results for 14 trials (Table 1). Of the trials with published results, 8 (57.1%) tested molnupiravir, 4 (28.6%) tested nirmatrelvir/ritonavir, and 2 (14.3%) tested both therapies. Five (35.7%) trials had a primary outcome of viral clearance, 4 (28.6%) trials had a primary outcome of hospitalization and death, 2 (14.3%) assessed COVID-19 symptoms, 2 (14.3%) assessed death, and 1 (7.1%) assessed hospitalization. All 3 terminated trials had published outcome data, indicating null results.

There were 4 trials that tested nirmatrelvir/ritonavir in long COVID. All used symptoms as a primary outcome for determining efficacy, and none had published results.

**Figure 2.**
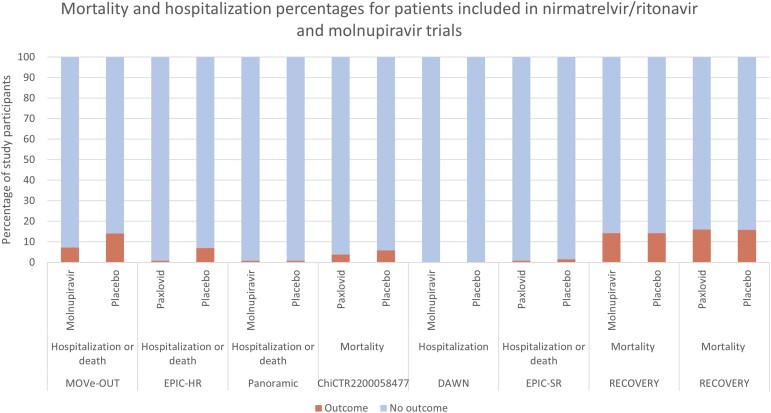
Percentages of patients with mortality and hospitalization outcomes in trials testing nirmatrelvir/ritonavir or molnupiravir against coronavirus disease 2019.

Three of the 4 trials that tested nirmatrelvir/ritonavir and reported death/hospitalization data failed to find a difference (Figure 2). The 1 trial that reported significant improvements predominantly tested people who were infected with earlier COVID-19 variants (eg, Delta), whereas the 3 null trials were tested in people with more recent variants. The 2 nirmatrelvir/ritonavir trials that used a primary outcome of symptoms that had reported results were negative. Two of the 5 trials that tested molnupiravir and reported death/hospitalization data found an improvement with molnupiravir. These 2 trials primarily included participants infected with the Delta variant, whereas the null trials were tested later, against more recent variants. The 1 trial with symptoms as a primary outcome that also had published results was negative.

**Figure 3.**
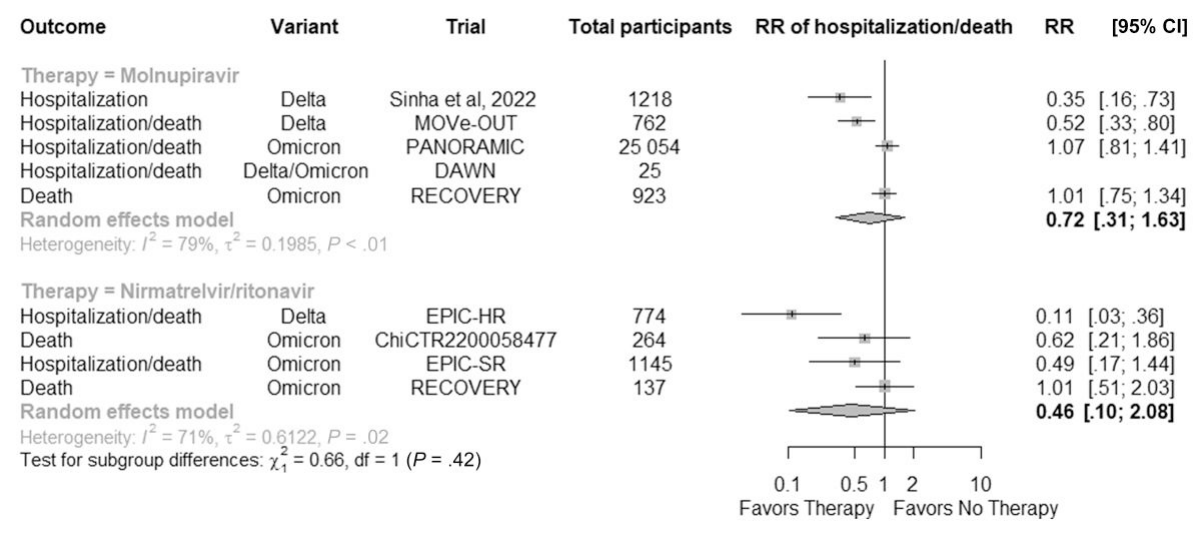
Forest plot of risk of hospitalization and/or death in trials testing nirmatrelvir/ritonavir or molnupiravir against coronavirus disease 2019. Abbreviations: CI, confidence interval; RR, relative risk.

Figure 3 shows the pooled risk of hospitalization and/or death in studies testing nirmatrelvir/ritonavir and molnupiravir, sorted by the date the study stopped accrual. There were 8 studies (1 that published data on both nirmatrelvir/ritonavir and molnupiravir) that had relevant information for the pooled estimate. A total of 27 982 participants were included in the molnupiravir trials, and 2320 participants were included in the nirmatrelvir/ritonavir trials. The pooled estimate for molnupiravir was 0.72 (95% CI, 0.31–1.63), and the pooled estimate for nirmatrelvir/ritonavir was 0.46 (95% CI, 0.10–2.08).

## DISCUSSION

We found 23 studies assessing nirmatrelvir/ritonavir and/or molnupiravir. Fourteen of these studies had published results; 2 of these trials reported favorable results for nirmatrelvir/ritonavir and 6 trials reported favorable results for molnupiravir (4 based on viral clearance). Many of the primary outcomes were based on viral clearance, rather than patient-centered outcomes, such as hospitalization or death, and most positive trials were tested against unvaccinated populations and/or earlier strains of SARS-CoV-2.

A notable finding from our study is the lack of benefit for hospitalization and death in the pooled analysis. The only study that reported improvements in these outcomes was the EPIC-HR study, which is unique in that it included unvaccinated and nonhospitalized individuals. Similarly, the MOVe-OUT trial reported improvements in hospitalizations and deaths in unvaccinated, nonhospitalized patients. Positive trials were also the only trials in the pooled analysis to be tested when the Delta variant was the most common variant. Other trials that included vaccinated individuals and tested against other strains did not find significant improvements with these therapies.

A number of studies (39%) evaluating these therapies have yet to report results, and considering the resources spent on testing and implementation of these therapies, it is important to be able to have all results when considering effectiveness. Results for the EPIC-SR study, a study that failed to find benefit with nirmatrelvir/ritonavir, are only available as press release data. Furthermore, the PANORAMIC study tested both nirmatrelvir/ritonavir and molnupiravir but has only published data on molnupiravir. According to ClinicalTrials.gov, 6 studies of the 23 (26%) are complete, and of the 6 completed studies, we found published data for only 3. These findings suggest that a full understanding of these drugs’ efficacy is incomplete. Moreover, because of publication bias, it is likely that many of the unpublished trials are or will be negative, and our results likely represent a better-case scenario than if all trials had reported data.

The efficacy of these antiviral therapies has been primarily demonstrated in trials tested when earlier strains, such as the Delta variant, were common. The lack of efficacy in more recent trials has not been discussed in the literature. It may be that there is little incentive to reevaluate effectiveness since these drugs already have approval. Regardless, as with all medical interventions, it is important to regularly assess the effectiveness of therapies to ensure that treatment is evidence-based and continues to provide patient benefit.

Several large, observational studies have been conducted, assessing the association between receipt of nirmatrelvir or molnupiravir and COVID-19 during the Omicron wave. Their findings indicate a striking reduction in deaths and hospitalization, suggesting possible efficacy. However, because of the observational nature of these studies, there is likely unaccounted confounding, which is most evident by the almost immediate separation of the survival curves between those who received nirmatrelvir or molnupiravir and those who did not [9, 10]. Indeed, other researchers have documented bias in these types of studies [11–13]. The randomized studies done during the Omicron wave have failed to corroborate any hospitalization or mortality benefit [6, 14, 15].

It is worth considering that these treatments may be beneficial to patients who are immunocompromised or have significant risk factors for progression to severe disease. This should be further tested in high-quality randomized controlled studies, although, it should be noted that 2 of the trials in this analysis included hospitalized patients, and both studies reported null findings [6, 14].

Despite declining effectiveness of these therapies, in terms of hospitalization and death, sales for nirmatrelvir/ritonavir and molnupiravir have continued to remain strong. The sales of nirmatrelvir/ritonavir are estimated to be $8 billion in 2023 and about $19 billion in 2022 [16]. For molnupiravir, sales are estimated to be about $4.7 billion in 2023 and $5.7 billion in 2022 [17]. Few cost-effectiveness analyses using randomized data have been done, but a recent analysis indicates that the use of molnupiravir is unlikely to be cost-effective in the general population, especially when vaccination rates are high [18]. To date, there have not been any analyses on nirmatrelvir/ritonavir. Considering increasing vaccination/seroprevalence rates and the results of cost-effectiveness studies, there may be better use of limited healthcare dollars.

Outcomes for determining effectiveness varied from serious outcomes, such as hospitalization and death, to ones less important for patients, such as viral clearance. Only 1 trial testing nirmatrelvir/ritonavir and 2 trials testing molnupiravir reported positive results for hospitalization and death, and no trial reported positive results when using the primary outcome of symptoms. Many of the positive trials used viral clearance as the primary outcome. And yet, there is low correlation between viral clearance and more serious outcomes, such as hospitalization, and likely any small correlation is limited to those who are unvaccinated and/or without prior infection [19].

Our analysis is limited by several factors. It is possible that we did not find all randomized studies evaluating the effectiveness of these therapies. To capture as many studies as possible, we not only used 3 databases, but we also searched ClincialTrials.gov to find additional studies. Second, we focused on hospitalization and death outcomes, rather than virus clearance, so our results may not reflect the findings at large. While we do include these studies in our analysis, we wanted to focus on outcomes most important to the health of patients and the population. Third, the incompleteness of the study publications may affect the generalizability of our findings.

In conclusion, the early data of nirmatrelvir/ritonavir and molnupiravir for the treatment of COVID-19 was positive, but trials during later strains of SARS-CoV-2 have failed to show clinical benefit, in terms of hospitalization and death. Against the backdrop of an evolving strain of virus and increasing population immunity, approval data for currently available treatments for COVID-19 need to be reevaluated, based on current strains and immunity.

## Supplementary Data

Supplementary materials are available at *Open Forum Infectious Diseases* online. Consisting of data provided by the authors to benefit the reader, the posted materials are not copyedited and are the sole responsibility of the authors, so questions or comments should be addressed to the corresponding author.

## Supplementary Material

ofae497_Supplementary_Data

## Notes

***Author contributions*.** A. H. and V. P. conceptualized study design. A. H. abstracted data and V. P. reviewed data. A. H. conducted data analysis and wrote the first draft of the manuscript. V. P. reviewed and finalized the manuscript.

***Financial support.*** This work was supported by ORMJMP.

***Data availability*.** We used publicly available data. Data for these analyses can be obtained upon request to the primary author.
