# Supplementary material for: A Systematic Review of Nirmatrelvir/Ritonavir and Molnupiravir for the Treatment of Coronavirus Disease 2019
Source: Open Forum Infect Dis. 2024 Sep 7;11(9):ofae497. doi: 10.1093/ofid/ofae497 (PMC11403474; doi:10.1093/ofid/ofae497)
Supplement: ofae497_Supplementary_Data [file ofae497_supplementary_data.zip › Paxlovid_PRISMA flowchart.docx]

Supplemental figure. Flowchart of selection of trials testing nirmatrelvir/ritonavir and molnupiravir for COVID-19 treatment

Records identified from*:

Nirmatrelvir/ritonavir

PubMed (n = 9)

Embase (n = 34)

Web of Science (n = 517)

Molnupiravir

PubMed (n = 16)

Embase (n = 34)

Web of Science (n = 59)

ClinicalTrials.gov (n = 93)

Duplicate records removed, excluding results from ClinicalTrials.gov (n = 85)

Duplicates from ClincalTrials.gov (n = 31)

**Identification**

Studies from included from PubMed/Embase/Web of Science

assessed for eligibility

(n = 584)

Clinical trials assessed for eligibility (n = 62)

Databases

Trials excluded (N=576):

Not an RCT (n =279)

Review/meta-analysis (n = 205)

Not in humans (n = 40)

Not testing a drug (n = 35)

Secondary analysis (n = 11)

Bioavailability (n = 3)

Other (n = 3)

ClinicalTrials.gov

Trials excluded (N=47):

Not an RCT (n = 13)

Not testing a drug (n = 11)

Duplicated in both searches (n = 5)

Not COVID (n = 8)

Bioavailability (n = 4)

Special population (n = 4)

Other (n = 2)

**Screening**

Studies included from PubMed/Embase/Web of Science

(n = 8)

Studies from ClinicalTrials.gov

(n = 15)

**Included**

Trials with published hospitalization/death results (n=14)

**With results – pooled analysis**
