# Supplementary material for: A Systematic Review of Nirmatrelvir/Ritonavir and Molnupiravir for the Treatment of Coronavirus Disease 2019
Source: Open Forum Infect Dis. 2024 Sep 7;11(9):ofae497. doi: 10.1093/ofid/ofae497 (PMC11403474; doi:10.1093/ofid/ofae497)
Supplement: ofae497_Supplementary_Data [file ofae497_supplementary_data.zip › supplemental table_paxlovid.docx]

Supplemental table. Trials testing nirmatrelvir/ritonavir and/or molnupiravir in patients with COVID-19.

| Registration number | Number of participants | Outcome | Study start and end dates | Country | Main variant | % Vaccinated | Trial Name | Status* | Results |
| --- | --- | --- | --- | --- | --- | --- | --- | --- | --- |
| Nirmatrelvir/ritonavir | | | | | | | | | |
| NCT04381936 | 137 | Death | 3/19/2020 – 11/2023 | Multiple | Omicron | 85 | RECOVERY | Ongoing | Null |
| NCT05601167 |  | Symptoms | 2/17/2021 - 6/1/2022 | Russia | NI |  |  | Completed | Unreported |
| NCT04960202 | 1039 | Hospitalization/  death | 7/16/2021 - 12/9/2021 | Multiple | Delta | 0 | EPIC-HR | Completed | Pos |
| NCT05011513 | 1153 | Symptoms | 8/25/2021 - 7/25/2022 | Multiple | Omicron |  | EPIC-SR | Terminated | Null |
| ISRCTN 30448031 | 26411 | Hospitalization/  death | 12/8/2021 - 4/27/2022 | UK | Omicron | 94 | PANORAMIC | Ongoing | Null |
| ChiCTR2200058477 | 264 | Death | 4/10/2022 - 5/19/2022 | China | Omicron | 26.52 |  | Ongoing | Null |
| NCT05041907 | 142 | Viral clearance | 6/6/2022 - 2/23/2023 | Thailand | Omicron | 100 | PLATCOV | Ongoing | Pos |
| NCT05576662** |  | Symptoms | 11/8/2022 - 8/14/2023 | US | Omicron |  | STOP-PASC | Completed | Unreported |
| NCT05614349 |  | Hospitalization/  death | 1/16/2023 – 1/2024 | Canada | NI |  | CanTreatCOVID | Ongoing | Unreported |
| NCT05823896** |  | Quality of life | 5/1/2023 -1/31/2024 | Sweden | NI |  | PROLIFIC | Ongoing | Unreported |
| NCT05852873** |  | Symptoms | 5/12/2023 – 4/2024 | Norway | NI |  | PanoramicNOR | Ongoing | Unreported |
| NCT05595369** | 923 | Symptoms | 7/26/2023 – 4/2024 | US | NI |  | RECOVER-VITAL | Ongoing | Null |
| NCT05668091** |  | Symptoms | 4/30/2024 -4/30/2024 | US | NI |  |  | Ongoing | Unreported |
| Molnupiravir | | | | | | | | | |
| NCT04405570 | 202 | Viral clearance | 6/19/2020 – 1/21/2021 | US | Alpha | 0 |  | Completed | Pos |
| NCT04575584 | 293 | Symptoms | 10/19/2020 – 1/12/2021 | multiple | Alpha | 0 | MOVE-IN | Terminated | Null |
| NCT04746183 | 180 | Viral clearance | 11/18/2020 - 3/16/2022 | UK | Delta | 50 | AGILE CST-2 | Ongoing | Null |
| NCT04575597 | 1433 | Hospitalization/  death | 5/6/2021 -10/2/2021 | Multiple | Delta | 0 | MOVe-OUT | Completed | Pos |
|  | 1218 | Hospitalization | 5/2021 – 8/2021 | India | Delta |  |  | Ongoing | Pos |
| NCT04730206 | 25 | Hospitalization/  death | 6/2021 – 7/2022 | Belgium | Delta/  omicron | 100 | DAWN | Terminated | Null |
| NCT05595824 |  | Symptoms | 12/1/2021 -3/11/2022 | Russia | NI |  |  | Completed | Unreported |
| ChiCTR2200056817 | 116 | Viral clearance | 3/3/2022 - 3/21/2022 | China | Omicron | 85.3 |  | Ongoing | Pos |
| ChiCTR2200059796 | 121 | Viral clearance | 3/26/2022 - 5/31/2022 | China | Omicron | 91.89 |  | Ongoing | Pos |
| NCT05041907 | 142 | Viral clearance | 6/6/2022 - 2/23/2023 | Thailand | Omicron | 100 | PLATCOV | Ongoing | Pos |
| NCT05459532 |  | Hospitalization/  death | 8/12/2022 – 7/2024 | South Africa | NI |  | CoTeT | Ongoing | Unreported |
| NCT04381936 | 923 | Death | 7/26/2023 – 6/2024 | Multiple | Omicron | 83 | RECOVERY | Ongoing | Null |

*Status according to clinicaltrials.gov.; **tested in patients with long COVID-19; NI=not indicated.
